# Less Social Participation Is Associated With a Higher Risk of Depressive Symptoms Among Chinese Older Adults: A Community-Based Longitudinal Prospective Cohort Study

**DOI:** 10.3389/fpubh.2022.781771

**Published:** 2022-02-09

**Authors:** Min Du, Wanwei Dai, Jue Liu, Jing Tao

**Affiliations:** ^1^Department of Epidemiology and Biostatistics, School of Public Health, Peking University, Beijing, China; ^2^Peking University Third Hospital, Beijing, China; ^3^Institute for Global Health and Development, Peking University, Beijing, China; ^4^National Health Commission Key Laboratory of Reproductive Health, Peking University, Beijing, China; ^5^College of Rehabilitation Medicine, Fujian University of Traditional Chinese Medicine, Fuzhou, China

**Keywords:** social participation, cohort, depressive symptoms, Chinese, older adults

## Abstract

**Objective:**

We aimed to examine the association between social participation and depressive symptoms among Chinese older people aged 65 years or above to supplement limited studies in China on this topic.

**Methods:**

This community-based longitudinal prospective cohort study used the data from the Chinese Longitudinal Healthy Longevity Survey (CLHLS, baseline in 2014 and a follow-up in 2018). Depressive symptoms were assessed using the 10-item Center for Epidemiologic Studies Depression Scale. Social participation was assessed using a composite index by considering the frequency for the two types of social activity: organized social activities and informal activities. Pearson's χ^2^ test was used to correlate the characteristics of participants with social participation or depressive symptoms. Log-binomial regression models were used to assess the association between social participation and the risk of depressive symptoms.

**Results:**

The incidence of depressive symptoms was 28.8% among 2,200 participants in 2018 after a 4-year follow-up. Participants with no social participation (32.6%), organized social activities (30.6%), or informal social activities (31.2%) were more likely to have depressive symptoms. After the adjustment of demographic factors, socioeconomic status, lifestyle habits, and health status, in comparison with older people who often engaged in social participation, organized social activities, and informal social activities, the risk of depressive symptoms was 45% [adjusted risk ratio (aRR): 1.45, 95% CI: 1.16–1.82], 42% (aRR: 1.45, 95% CI: 1.02–2.00), and 29% (aRR: 1.29, 95% CI: 1.02–1.99) higher among older people with no social participation and who never engaged in organized social activities and informal social activities, respectively.

**Conclusions:**

This study showed that the lack of social participation, including organized social activities and informal social activities, was associated with a higher risk of depressive symptoms after 4 years among older adults in China. Our findings shed lights into the feasibility of promoting social participation to reduce the risk of depressive symptoms and promote longevity and healthy aging among older adults.

## Introduction

Aging is a great challenge to countries worldwide, including China (1, 2). According to the 2020 China Statistical Yearbook, there were 176 million people aged 65 years or above in the country, accounting for 12.6% of the total population in China (2). The issue of aging has broad global health implications in terms of cancer, cognitive impairment, depressive disorders, etc. (3). Depression is the most widespread mental disorder, affecting more than 264 million people globally (4). Depression is prevalent among older people. According to the report *Depression and Other Common Mental Disorders Global Health Estimates* from the WHO in 2017, the prevalence of depression is nearly 5% among older people, plateauing among women aged 55–74 years at more than 7.5%, in comparison with age groups 15–39, 44–54, and 75 years or above (all below 7.5%) (4). Additionally, that report showed that the estimated number of people living with depression increased by 18.4% between 2005 and 2015, which reflects a proportionate increase among older adults (4). In China, Tan et al. reported that the prevalence of depression was 15.94% among 19,420 older people in 2016 (5). Ning et al. reported that 27% of 2,410 Chinese older adults (age ≥ 60 years) had a depression in 2019 (6). Generally speaking, a larger number of people with depression live in Southeast Asia and the Western Pacific Region, especially in India and China (4, 5). Depression is a leading cause of disability around the world and is ranked the single largest contributor to nonfatal health loss (7.5% of all years lived with disability) (4). Additionally, depression is associated with heart-related disorders and peripheral/vascular-related disorders (7). Therefore, to promote longevity and healthy aging, exploring the influencing factors of depression and reducing the prevalence of this disorder are crucial and urgently needed among older adults.

In the context of the disease burden owing to depression among older people in China, behavioral interventions might have tangible benefits for health and wellbeing at older ages. To date, studies have shown that dietary factors (less alcohol consumption and more tea consumption), sufficient sleep, and more social participation have key roles in preventing depression among older adults (5, 8). Social participation is defined as the involvement of people in activities either voluntarily or mandatorily in formal or informal social groups (9). Social participation builds resilience owing to the social support received, and resilience is critical for maintaining independence and facilitating active aging (10). Previous studies have found that social participation is associated with mortality, disability, and cognitive impairment; however, related research on the association between social participation and depression is relatively limited in China. Cross-sectional studies have been conducted in Brazil (11), Canada (12), Germany (13), the USA (14), South Africa (15), and some provinces of China (6, 16, 17) to show that social participation is associated with depression. However, a cross-sectional design limits the ability to infer causality or the depression leading to a lower degree of social participation (18, 19); therefore, cohort studies are needed to estimate this effect. Several cohort studies have reported that social participation has a protective effect against depression among older adults in Japan (20–23), Korea (24), and the USA (25). However, the results have been inconsistent, and studies in China (26, 27) and the UK (23) have found no relationship between social participation and depression. Previous studies have shown that smoking (28), sleep (5, 29), and body mass index (BMI) (5) are associated with depression. Tomioka et al. found that social participation is associated with self-rated health (30). Although most studies were mainly controlled for demographics (e.g., age, sex, and marital status) and health status (e.g., chronic diseases, self-perceived health, and physical function), lifestyle habits (e.g., smoking status), and health status (e.g., BMI, sleep quality, and self-perceived quality of life) as the possible confounding factors were not well controlled. Furthermore, there is a lack of cohort studies in China to explore the effect of social participation on depression after controlling for demographic characteristics, lifestyle habits, and health status.

China is facing challenges owing to its population aging, and Chinese older people have a high disease burden owing to depression (2, 4, 5). Half of the middle-aged and older people are not involved in social activities in China (31). Exploring the relationship between social participation and depression in cohort studies is vital for reducing the risk of depression in China. The Chinese Longitudinal Healthy Longevity Survey (CLHLS) is a nationally representative population-based survey conducted in 23 of 31 provinces in China. Owing to the information regarding the older population, including demographics, lifestyle, and health status, this survey is commonly used in aging research in China (32). Previous cohort studies have mainly considered the association of one type or a composite index of social participation with depression (23–25), and studies on the association between the specific types of social participation and depression are scarce. Considering the inconsistent results, an insufficient control for confounders, and limited cohort studies among Chinese older adults, in this study, we aimed to examine the association between social participation and depressive symptoms in Chinese adults aged 65 years or more using the data from the CLHLS.

## Methods

### Participants and Procedure

We used the data from the CLHLS, which is an ongoing, prospective cohort study that covers 23 of 31 provinces in China. This study was established in 1998, with a subsequent follow-up and recruitment of new participants in 2000, 2002, 2005, 2008, 2011, 2014, and 2018. The details of this study have been described elsewhere (32). The present analysis included the data from the eighth wave in 2014 (at baseline) and the latest wave in 2018. This study was approved by the Research Ethics Committee of Peking University (IRB00001052-13074). All participants or their legal representative signed a written consent form agreeing to participate in the baseline and follow-up surveys.

**Figure 1:**
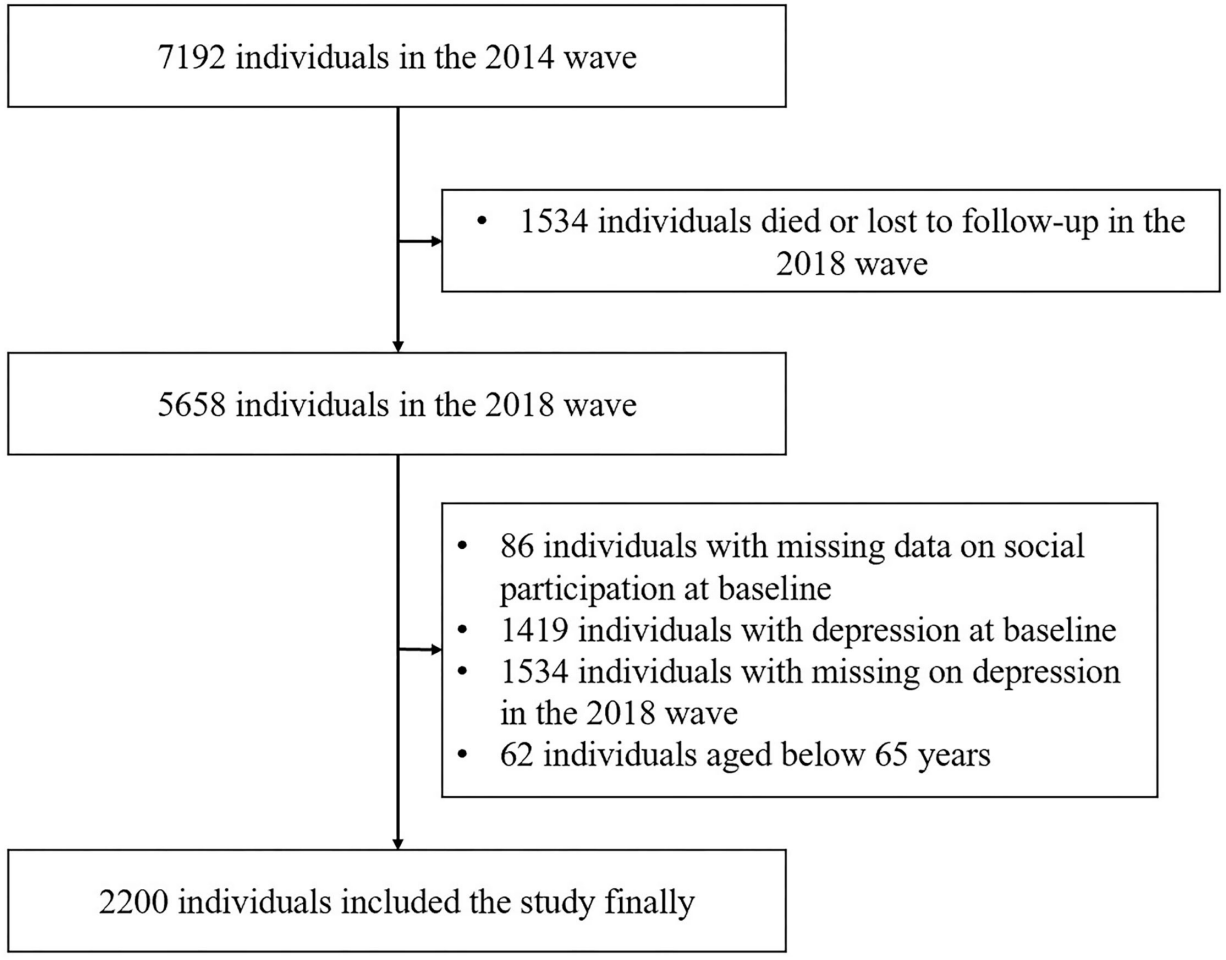
Flowchart of participant selection.

The present study is a longitudinal prospective cohort, an epidemiological study design that enables us to infer causality (33, 34). The 2014 survey wave included 7,192 Chinese older individuals. We excluded 1,534 individuals who either died or were lost to a follow-up, 86 individuals who had missing data on social participation in 2014, and 1,419 individuals with depressive symptoms in 2014. We collected the information on the incidence of depressive symptoms in 2018 and then excluded 1,534 individuals with missing data on depressive symptoms in 2018 and 62 individuals aged less than 65 years. For an analysis of the association between social participation and depressive symptoms, finally, in this study, we included 2,200 participants in the final analysis. Figure 1 shows the full process of inclusion and exclusion of research participants in this study.

### Assessment of Social Participation

Deng et al. defined social participation using two aspects, organized activities and informal activities (35). In our study, participants' social participation was assessed using a composite index that was the sum of these two types of social activity and involved activities such as playing cards or mahjong. Survey questions “Do you now perform social activities (organized) regularly?” and “Do you now perform the following activities (playing cards or mahjong) regularly?” were asked to assess organized and informal activities, respectively. The response options for both questions were “almost every day,” “not every day but at least once in a week,” “not every week but once a month,” “not every month but sometimes,” and “never.” We defined the option “not every month but sometimes” as “sometimes” and the remaining options (except “never”) as “often.” Participants who responded “often” for any one type of activity were included in the “often” group; those who responded “never” for both types of activity were included in the “never” group; the remaining individuals were included in the “sometimes” group. The frequencies of social participation and engaging in organized social activities and informal activities were the independent variables.

### Measurement of Depressive Symptoms

The dependent variables were depressive symptoms measured using the 10-item Center for Epidemiologic Studies Depression Scale (CES-D-10) (36). This scale is a widely used survey tool to measure depressive symptoms among Chinese older adults, with good validity and reliability (8, 27, 37–40). All items are rated on a four-point scale, from “rarely” to “on some days” (1–2 days), “occasionally” (3–4 days), or “most of the time” (5–7 days). We scored the responses of “rarely,” “on some days” (1–2 days), “occasionally” (3–4 days), and “most of the time” (5–7 days) as 0, 1, 2, and 3, respectively, after we reversely coded the responses to two positive questions—“I was happy” and “I felt hopeful about the future.” The total range of CES-D-10 scores is 0–30, with higher scores indicating a greater severity of depressive symptoms. The well-validated cutoff value is 10 in measuring depressive symptoms among Chinese older populations (8, 37); therefore, participants with a score ≥ 10 on the CES-D-10 were considered to have depressive symptoms.

### Covariates

The control variables included demographic factors, socioeconomic status, lifestyle habits, and health status. Demographic factors included age (continuous age in years and categorical age groups: 65–79/≥80 years), sex (male/female), residential area (urban/rural), marital status (married/unmarried, divorced, or widowed), and living status (living alone or not). Socioeconomic status included education level (no formal schooling/at least 1 year of education) and household income (low/moderate/high), which was classified based on tertiles. Lifestyle habits included smoking status (never/previous/current) and consuming alcohol status (never/previous/current). Health status was measured using BMI (underweight/normal/overweight/obese), sleep quality (good/moderate/poor), functional disability (yes/no), self-perceived quality of life (good/moderate/poor), self-perceived health (good/moderate/poor), and the number of chronic diseases (hypertension, diabetes, heart disease, stroke, bronchitis, and cancer; 0/1/≥2). According to WHO cutoff points, BMI was categorized as underweight (<18.5 kg/m^2^), normal (18.5–24.9 kg/m^2^), overweight (25–29.9 kg/m^2^), and obese (≥30 kg/m^2^). Functional disability refered to basic personal care tasks of daily life, which was defined as self-reported difficulty with any one of the following activities of daily living (ADL): dressing, eating, bathing, continence, toileting and cleaning, and indoor movement (41). Sleep quality, self-perceived quality of life, and self-perceived health were assessed using the questions “How is the quality of your sleep?,” “How do you rate your life at present?,” and “How do you rate your health at present?,” respectively. Response options to all these questions were “very good,” “good,” “so-so,” “poor,” and “very poor.” We classified participants who responded “very good” and “good” as the “good” group, those who responded “so-so” as the “moderate” group, and those who responded “poor” and “very poor” as the “poor” group.

### Data Analysis

Baseline characteristics of the study population are described as percentages for categorical variables. Pearson's χ^2^ test was used to correlate the characteristics of participants with social participation or depressive symptoms. We used a log-binomial regression model to assess the association between social participation and depressive symptoms, which is an appropriate analysis for cohort data with a relatively high incidence of outcome (42). We conducted a sensitivity analysis by fitting the different models to examine the robustness of the estimation. Model 1 was a univariate model. We adjusted the demographic factors and socioeconomic status in model 2, including age, sex, residential area, marital status, living status, education level, and household income. We adjusted all covariates in the adjusted model 3, including age, sex, residential area, marital status, living status, education level, household income, smoking status, consuming alcohol status, BMI, sleep quality, functional disability, self-perceived quality of life, self-perceived health, and the number of chronic diseases. We calculated the crude risk ratios (cRRs) and adjusted risk ratios (aRRs) with 95% CIs for the effect of social participation on depressive symptoms. To assess whether the different modes of social participation had different effects on depressive symptoms, we also used models of the association between organized social activities/informal activities and depressive symptoms. Additionally, we analyzed the relationship of social participation with the original five frequencies including “almost every day,” “not every day but at least once in a week,” “not every week but once in a month,” “not every month but sometimes,” and “never” on depressive symptoms. To examine the robustness of the estimation, we conducted a sensitivity analysis by replacing categorical variables with continuous variables, including age, education level, household income, and ADL scores. The value of *p* < 0.05 was used to indicate statistical significance. All analyses were performed with IBM SPSS 26.0 (IBM Corp., Armonk, NY, USA).

## Results

### Social Participation

Baseline characteristics of the 2,200 participants are shown in Table 1. In total, 54.5% of the participants were 65–79 years old and 52.7% of the participants were men. Overall, 63.4% of the participants never had social participation at baseline. Participants who were more likely to have no social participation were aged ≥ 80 years, women, living in a rural area, unmarried/divorced/widowed, living alone, illiterate, underweight, those who never smoked or consumed alcohol, and had low household income, functional disability, poor self-perceived quality of life, and self-perceived health (all *p* < 0.05, Table 1). Social participation was not associated with sleep quality and the number of chronic diseases (all *p* > 0.05).

**Table 1:** Social participation by demographic factors, socioeconomic status, lifestyle habits, and health status.

**Variable**	***N***	**Social participation**			**X^2^**	***P*** **-value**
		**Never**	**Sometimes**	**Often**		
**Total**	2,200	1,395 (63.4)	214 (9.7)	591 (26.9)		
**Demographic factors**					
Age group (years)					73.191	<0.001*
65–79	1,198	664 (55.4)	148 (12.4)	386 (32.2)		
≥80	1,002	731 (73.0)	66 (6.6)	205 (20.5)		
Sex					57.224	<0.001*
Male	1,159	650 (56.1)	140 (12.1)	369 (31.8)		
Female	1,041	745 (71.6)	74 (7.1)	222 (21.3)		
Residential area					17.408	<0.001*
Urban	949	560 (59.0)	115 (12.1)	274 (28.9)		
Rural	1,251	835 (66.7)	99 (7.9)	317 (25.3)		
Marital status					33.696	<0.001*
Married	1,221	710 (58.1)	142 (11.6)	369 (30.2)		
Unmarried, divorced, or widowed	979	685 (70.0)	72 (7.4)	222 (22.7)		
Living alone					10.604	0.005*
Yes	472	329 (69.7)	35 (7.4)	108 (22.9)		
No	1,728	1,066 (61.7)	179 (10.4)	483 (28.0)		
**Socioeconomic status**					
Educational level (years)					135.627	<0.001*
No formal schooling	989	758 (76.6)	61 (6.2)	170 (17.2)		
At least 1 year of education	1,211	637 (52.6)	153 (12.6)	421 (34.8)		
Household income (tertiles)					47.630	<0.001*
Low	711	509 (71.6)	45 (6.3)	157 (22.1)		
Moderate	831	530 (63.8)	82 (9.9)	219 (26.4)		
High	658	356 (54.1)	87 (13.2)	215 (32.7)		
**Lifestyle habits**						
Smoking status					61.137	<0.001*
Never	1,484	1,022 (68.9)	128 (8.6)	334 (22.5)		
Previous	275	149 (54.2)	37 (13.5)	89 (32.4)		
Current	434	221 (50.9)	49 (11.3)	164 (37.8)		
Consuming alcohol					45.372	<0.001*
Never	1,559	1,051 (67.4)	138 (8.9)	370 (23.7)		
Previous	213	125 (58.7)	20 (9.4)	68 (31.9)		
Current	410	205 (50.0)	55 (13.4)	150 (36.6)		
**Health status**						
Body mass index (kg/m^2^)					15.122	0.019*
Underweight	270	195 (72.2)	21 (7.8)	54 (20.0)		
Normal	1,463	929 (63.5)	141 (9.6)	393 (26.9)		
Overweight	391	226 (57.8)	43 (11.0)	122 (31.2)		
Obese	76	45 (59.2)	9 (11.8)	22 (28.9)		
Sleep quality					7.578	0.108
Good	1,432	884 (61.7)	146 (10.2)	402 (28.1)		
Moderate	540	366 (67.8)	42 (7.8)	132 (24.4)		
Poor	223	143 (64.1)	25 (11.2)	55 (24.7)		
Functional disability					10.632	0.005*
No	1,984	1,237 (62.3)	202 (10.2)	545 (27.5)		
Yes	216	158 (73.1)	12 (5.6)	46 (21.3)		
Self-perceived quality of life					15.867	0.003*
Good	1,552	945 (60.9)	168 (10.8)	439 (28.3)		
Moderate	612	424 (69.3)	43 (7.0)	145 (23.7)		
Poor	35	25 (71.4)	3 (8.6)	7 (20.0)		
Self-perceived health					38.226	<0.001*
Good	1,168	684 (58.6)	116 (9.9)	368 (31.5)		
Moderate	822	550 (66.9)	78 (9.5)	194 (23.6)		
Poor	207	159 (76.8)	20 (9.7)	28 (13.5)		
Number of chronic diseases					2.196	0.700
0	1,057	672 (63.6)	105 (9.9)	280 (26.5)		
1	622	392 (63.0)	66 (10.6)	164 (26.4)		
≥2	521	331 (63.5)	43 (8.3)	147 (28.2)		

* *Missing values: smoking status (n = 7; 0.32%), drinking status (n = 18; 0.82%), sleep quality (n = 5; 0.23%), self-perceived quality of life (n = 1; 0.05%); self-perceived health (n = 3; 0.14%); p < 0.05. Values in the table are n (%) unless otherwise stated*.

### Depressive Symptoms

The incidence of depressive symptoms was 28.8% among 2,200 participants in 2018 after a 4-year follow-up. Older individuals who never had social participation or engaged in organized social activities and informal social activities were more likely to have depressive symptoms. Additionally, participants who were aged ≥80 years, women, living in a rural area, unmarried/divorced/widowed, living alone, illiterate, those who never smoked or consumed alcohol, and who had low household income, poor sleep quality, poor self-perceived quality of life, poor self-perceived health, and more than two chronic diseases were more likely to have depressive symptoms (all *p* < 0.05, Table 2). However, depressive symptoms were not associated with BMI and functional disability (*p* > 0.05).

**Table 2:** The incidence of depressive symptoms among Chinese older adults by social participation status, demographic factors, socioeconomic status, lifestyle habits, and health status.

**Variable**	**Depressive symptoms**			**X^2^**	***P*** **-value**
	**Total**	***N***	**Prevalence (%)**		
**Total**	2,200	634	28.8		
**Social participation**					
Social participation					
Never	1,395	455	32.6	28.519	<0.001*
Sometimes	214	55	25.7		
Often	591	124	21.0		
**Modes of social participation**					
Organized social activities				15.757	<0.001*
Never	1,766	541	30.6		
Sometimes	203	49	24.1		
Often	231	44	19.0		
Informal social activities				18.042	<0.001*
Never	1,637	510	31.2		
Sometimes	112	29	25.9		
Often	451	95	21.1		
**Demographic factors**					
Age group (years)				11.735	0.001*
65–79	1,198	309	25.8		
≥80	1,002	325	32.4		
Sex				16.438	<0.001*
Male	1,159	291	25.1		
Female	1,041	343	32.9		
Residential area				4.170	0.041*
Urban	949	252	26.6		
Rural	1,251	382	30.5		
Marital status				11.544	0.001*
Married	1,221	316	25.9		
Unmarried, divorced or widowed	979	318	32.5		
Living alone				8.204	0.004*
Yes	472	161	34.1		
No	1,728	473	27.4		
**Socioeconomic status**					
Educational level (years)				58.734	<0.001*
No formal schooling	989	366	37.0		
At least 1 year of education	1,211	268	22.1		
Household income, tertiles				42.277	<0.001*
Low	711	262	36.8		
Moderate	831	234	28.2		
High	658	138	21.0		
**Lifestyle habits**					
Smoking status				14.247	0.001*
Never	1,484	465	31.3		
Previous	275	63	22.9		
Current	434	104	24.0		
Consuming alcohol				6.049	0.049*
Never	1,559	472	30.3		
Previous	213	55	25.8		
Current	410	101	24.6		
**Health status**					
Body mass index (kg/m^2^)				4.851	0.183
Underweight	270	91	33.7		
Normal	1,463	420	28.7		
Overweight	391	101	25.8		
Obese	76	22	28.9		
Sleep quality				48.132	<0.001*
Good	1,432	344	24.0		
Moderate	540	195	36.1		
Poor	223	93	41.7		
Functional disability				0.828	0.363
No	1,984	566	28.5		
Yes	216	68	31.5		
Self-perceived quality of life				42.788	<0.001*
Good	1,552	396	25.5		
Moderate	612	214	35.0		
Poor	35	23	65.7		
Self-perceived health				54.955	<0.001*
Good	1,168	267	22.9		
Moderate	822	271	33.0		
Poor	207	94	45.4		
Number of chronic diseases				9.786	0.007*
0	1,057	279	26.4		
1	622	178	28.6		
≥2	521	177	34.0		

* *p < 0.05*.

### Association Between Social Participation and Depressive Symptoms

In the univariate model, older people who never had social participation or engaged in organized social activities and informal social activities had a higher risk of depressive symptoms (all *p* < 0.05). After adjusting for demographic factors and socioeconomic status including age, sex, residential area, marital status, living status, education level, and household income, the association remained significant (all *p* < 0.05). In the multivariable models, after the adjustment of all covariates, compared with older people who had frequent social participation, organized social activities, or informal social activities, the risk of depressive symptoms was 45% (aRR: 1.45, 95% CI: 1.16–1.82), 42% (aRR: 1.45, 95% CI: 1.02–2.00), and 29% (aRR: 1.29, 95% CI: 1.02–1.99) higher among older people who never had social participation or engaged organized social activities, or informal social activities, respectively (Table 3). The analysis of the relationship between social participation with the original five frequencies and depressive symptoms showed the similar results (Supplementary Table S1). In addition, a sensitivity analysis in which we included categorical variables as continuous variables showed the similar results.

**Table 3:** Association between social participation and the risk of depressive symptoms in univariate and multivariate models.

	**Depressive symptoms**					
	**Model A^a^**		**Model B^b^**		**Model C^c^**	
	**cRR (95% CI)**	***P*** **-value**	**aRR (95% CI)**	***P*** **-value**	**aRR (95% CI)**	***P*** **-value**
**Social participation**
Social participation
Never	1.53 (1.22–1.92)	<0.01*	1.44 (1.15–1.81)	<0.01*	1.45 (1.16–1.82)	<0.01*
Sometimes	1.22 (0.85–1.75)	0.24	1.24 (0.87–1.78)	0.24	1.26 (0.88–1.79)	0.21
Often	1 (reference)		1 (reference)		1 (reference)	
**Types of social participation**
Organized social activities
Never	1.53 (1.08–2.18)	0.02*	1.49 (1.04–2.12)	0.03*	1.42 (1.02–2.00)	0.04*
Sometimes	1.26 (0.80–2.00)	0.32	1.30 (0.82–2.07)	0.26	1.28 (0.82–2.01)	0.28
Often	1 (reference)		1 (reference)		1 (reference)	
Informal social activities
Never	1.36 (1.06–1.74)	0.02*	1.33 (1.04–1.71)	0.03*	1.29 (1.02–1.66)	0.04*
Sometimes	1.18 (0.74–1.90)	0.49	1.22 (0.76–1.94)	0.41	1.18 (0.74–1.90)	0.49
Often	1 (reference)		1 (reference)		1 (reference)	

* *p < 0.05*.

*cRR, crude risk ratio; aRR, adjusted risk ratio; CI, confidence interval*.

a *Model A: Univariate model*.

b *Model B: Adjusted for demographic factors and socioeconomic status*.

c *Model C: Adjusted for demographic factors, socioeconomic status, lifestyle habits, and health status*.

## Discussion

In our prospective national cohort study, the incidence of depressive symptoms was 28.8% among 2,200 Chinese older individuals in 2018 after a 4-year follow-up. In total, 63.4% of participants never had social participation. More importantly, we found that older people who never engaged in social participation, organized social activities, or informal social activities had a significantly increased risk of depressive symptoms, after the adjustment for age, sex, residential area, marital status, living status, education level, household income, smoking status, consuming alcohol status, BMI, sleep quality, functional disability, self-perceived quality of life, self-perceived health, and the number of chronic diseases.

Although some studies have found no relationship between social participation and depressive symptoms (23, 26, 27), our results were in accordance with the findings of the published cohort studies (20). A nationwide prospective cohort study among Japanese older adults found that social participation was associated with a lower prevalence of depressive symptoms using the Geriatric Depression Scale (20). Nakagomi et al. reported that community-level civic participation was associated with lower depressive symptoms among functionally independent adults aged 65 years or older in Japan (21). Nakagomi et al. further analyzed the impact of social participation on depressive symptoms stratified by sex and found that social participation is related to depressive symptoms in both men and women (21). In China, Liu et al. showed that social participation could ameliorate depressive symptoms after controlling for age, sex, and marital status among middle-aged and older participants (40). They also emphasized that in older migrants, more social participation could decrease depressive symptoms (40). Our study was conducted in a large study area including 23 research locations in 23 provinces of mainland China. We controlled basic demographic characters, lifestyle habits, health status, and socioeconomic status, which are related to depressive symptoms and social participation. We found that after adjusting for all covariates, compared with older people who had frequent social participation, those with no social participation had a 45% higher risk of depressive symptoms. Previous cohort studies have mainly considered the association of one type or a composite index of social participation with depression (23–25). We further analyzed the association of different types of social participation with depressive symptoms. Compared with older people who frequently engaged in organized social activities or informal social activities, the risk of depressive symptoms was 42 and 29% higher among older people who never participated in organized social activities or informal social activities, respectively, that is to say, given the protective effects for older people, social participation, including organized social activities and informal social activities, seems to be particularly well suited for this population to reduce the risk of depressive symptoms. More social participation may moderate depressive symptoms by increasing close social ties and social contact with neighbors (27, 43), as well as providing emotional social support (24). Noguchi et al. included social participation as an indicator of social isolation and found that social isolation was related to depressive symptoms; however, social participation was not related to depressive symptoms (23). Choi et al. found that emotional social support is a crucial mechanism by which social participation reduces the risk of depressive symptoms (24). They further reported that economic activity predicts lower levels of emotional social support, meaning that individuals engaged in economic activity may have fewer close social ties and less social contact with others, which may preclude the development of high-quality relationships (24, 27, 43). Therefore, it is necessary to analyze the impact of the different types of social participation on depression. Additionally, the specific biological mechanism in this association remains unclear and should be explored in the future. Considering the negative effect of not engaging in social participation in terms of depressive symptoms, encouraging older people to participate in organized and informal social activities to reduce their risk of depressive symptoms is important and urgently needed. To date, previous studies have investigated social participation using survey data because there is no standard measurement tool for social participation. Social participation is defined as the involvement of people in community activities either voluntary or mandatory, or formal and informal social groups (9). Guo et al. operationalized social participation as the four items that included visiting friends or family; attending religious services; participating in clubs, classes, or other organized activities; and going out for enjoyment (25). Liu et al. measured social participation using six items, including interacting with friends, playing games like mahjong, and providing help to family or friends (40). Croezen et al. (44) investigated social participation by determining whether individuals had engaged in the following activities during the previous month: voluntary or charity work; educational or training courses; sports, social clubs, or any other kinds of club activities; participation in religious organizations; and engagement with political or community organizations. Noguchi et al. evaluated social participation as having any or no social participation (no participation in any social or religious groups) (23). Owing to differences in the investigation of social participation, comparisons of the association between social participation and depressive symptoms may be limited. Therefore, a standard tool with high reliability and validity for investigating social participation must be developed in the future.

In our study, the incidence of depressive symptoms was 28.8% among 2,200 participants in 2018 after a 4-year follow-up, which was in line with the results of other studies. Ning et al. reported that 27% of Chinese older adults (age ≥ 60 years) had depressive symptoms in 2019 (6). The incidence of clinically significant depressive symptoms is 25.75% in Mexico older adults (45). The average prevalence of depressive symptoms among Japanese older adults was 28.6% in 2010 and 21.3% in 2016 (22). Because many older people must care for a spouse who is ill (38), are transitioning to retirement (46), or have experienced the death of a spouse (47), these individuals can easily become depressed. We found that Chinese older people who were women, living in rural areas, unmarried/divorced/widowed, living alone, or who had poor self-perceived health and more than two chronic diseases were more likely to be depressed, which is in line with other studies (6, 21, 23). Additionally, participants who were aged ≥80 years and illiterate, never smoked or consumed alcohol, and had low household income, poor sleep quality, and poor self-perceived quality of life were more likely to have depressive symptoms. Our findings provide a reference for key demographic details in the management of and improving depressive symptoms.

According to Peng et al., half of the middle-aged and older people in China are not involved in social activities (31). In our study, 63.4% of participants never had social participation. Moreover, older participants who lived alone and were illiterate were more likely to have no social participation. Barrenetxea et al. also reported that living alone [odds ratio (OR) = 1.93, 95% CI: 1.58–2.35] and lower education levels (no formal education: OR = 2.91, 95% CI: 2.35–2.60) were associated with a higher risk of social disconnection (48). We found that participants who were aged ≥80 years, women, living in rural areas, unmarried/divorced/widowed, and had low household income were more likely to have no social participation. Social participation is not only associated with depressive symptoms but also with mortality, disability, and cognitive impairment (12, 31, 49–52). Therefore, strategies, including peer-based interventions (53), social networking sites (54), and integrated resource utilization in the community (55, 56), should be reinforced to increase accessibility to social activities and enrich the available types of social participation.

A major strength of this study was that we estimated the impact of social participation on depressive symptoms after controlling for demographic factors, socioeconomic status, lifestyle habits, and health status among Chinese older people in a national cohort study. There are also several limitations in our study. We did not control or assess whether changes in, or the duration of, social participation affect depressive symptoms among older people. We estimated the effect of different types of social participation on depressive symptoms; however, more specific types of social participation, including group meetings and working with neighbors, were not investigated in detail in the CLHLS. Finally, in our study, among 7,192 individuals at baseline, 1,534 either died or were lost to a follow-up in 2018; therefore, we could not assess depressive symptoms in these individuals, and thus, we may have underestimated depressive symptoms in those who died.

In conclusion, our nationwide longitudinal prospective cohort study showed that less social participation, including organized social activities and informal social activities, may have a negative impact on depressive symptoms in Chinese older adults. Our findings suggested that, to achieve longevity and healthy aging among older adults, promoting social participation to reduce the risk of depressive symptoms may be feasible in China.

## Data Availability Statement

Publicly available datasets were analyzed in this study. Data are from the Chinese Longitudinal Healthy Longevity Survey 2014–2018 which is a public, open access repository (https://opendata.pku.edu.cn).

## Ethics Statement

The CLHLS was approved by the Ethical Review Committee of Peking University (IRB00001052-13074). All the participants signed an informed consent at the time of participation. The research was performed in accordance with the Declaration of Helsinki. The patients/participants provided their written informed consent to participate in this study.

## Author Contributions

JL: conceptualization, funding acquisition, and supervision. MD, WD, and JL: formal analysis. MD and WD: writing—original draft. MD, WD, JT, and JL: writing—review and editing. All authors have made substantial contributions to the conception, and the design of the work; or the acquisition, analysis, or interpretation of data for the work. All authors participated in drafting the manuscript and approved the published version.

## Funding

This study was supported by the grants of National Natural Science Foundation of China (Nos. 72122001 and 71934002), National Key Research and Development Project of China (Nos. 2021ZD0114101, 2021ZD0114104, and 2021ZD0114105), and National Statistical Science Research Project (No. 2021LY038). The funding body had no role in the design or conduct of the study; the collection, management, analysis, or interpretation of the data; the preparation, review, or approval of the manuscript; or the decision to submit the manuscript for publication.

## Conflict of Interest

The authors declare that the research was conducted in the absence of any commercial or financial relationships that could be construed as a potential conflict of interest.

## Publisher's Note

All claims expressed in this article are solely those of the authors and do not necessarily represent those of their affiliated organizations, or those of the publisher, the editors and the reviewers. Any product that may be evaluated in this article, or claim that may be made by its manufacturer, is not guaranteed or endorsed by the publisher.
