# Supplementary material for: Less Social Participation Is Associated With a Higher Risk of Depressive Symptoms Among Chinese Older Adults: A Community-Based Longitudinal Prospective Cohort Study
Source: Front Public Health. 2022 Feb 9;10:781771. doi: 10.3389/fpubh.2022.781771 (PMC8863664; doi:10.3389/fpubh.2022.781771)
Supplement: Supplementary file 1 [file Table_1.DOCX]

**Supplemental Table 1. Association between social participation and the risk of depressive symptoms in multivariate models**

|  | **aRR (95% CI)** | **p value** |
| --- | --- | --- |
| **Social participation** |  |  |
| Social participation |  |  |
| Never | 1.50 (1.12-2.01) | 0.007* |
| Not every month but sometimes | 1.53 (1.08-2.15) | 0.015* |
| Not every week but once in a month | 1.22 (0.80-1.86) | 0.36 |
| Not every day but at least once in a week | 1.40 (0.95-2.06) | 0.09 |
| Almost every day | 1 (reference) |  |
| **Modes of social participation** |  |  |
| Organized social activities |  |  |
| Never | 1.42 (0.94-2.16) | 0.09 |
| Not every month but sometimes | 1.46 (0.92-2.34) | 0.11 |
| Not every week but once in a month | 1.25 (0.70-2.26) | 0.45 |
| Not every day but at least once in a week | 0.98 (0.51-1.88) | 0.95 |
| Almost every day | 1 (reference) |  |
| Informal social activities |  |  |
| Never | 1.41 (1.05-1.91) | 0.024* |
| Not every month but sometimes | 1.44 (0.95-2.20) | 0.08 |
| Not every week but once in a month | 1.24 (0.77-2.00) | 0.38 |
| Not every day but at least once in a week | 1.46 (0.98-2.17) | 0.06 |
| Almost every day | 1 (reference) |  |

*p<0.05; aRR, adjusted risk ratio. Model adjusted for demographic factors, socioeconomic status, lifestyle habits, and health status.
